# Takotsubo cardiomyopathy associated with *Protobothrops mucrosquamatus* envenomation: a case report

**DOI:** 10.3389/fcvm.2025.1598373

**Published:** 2025-06-03

**Authors:** HanDong Wu, KeChun Zhou, XiaoTong Ma

**Affiliations:** Department of Emergency Medicine, The Fifth Affiliated Hospital of Wenzhou Medical University, Lishui Central Hospital, Lishui Hospital of Zhejiang University, Lishui, Zhejiang, China

**Keywords:** Takotsubo cardiomyopathy (TTS), *Protobothrops mucrosquamatus*, echocardiography, coronary angiography, fractional flow reserve (FFR), snake venom

## Abstract

**Background:**

*Protobothrops mucrosquamatus*, commonly known as the Taiwan habu, is a highly venomous snake species. Its venom is rich in haemotoxins and neurotoxins, capable of inducing severe coagulopathy, tissue necrosis, and multi-organ damage. However, to date, there have been no reported cases of Takotsubo cardiomyopathy (TTS) triggered by envenomation from *P. mucrosquamatus*. TTS is characterised by transient left ventricular dysfunction precipitated by acute stress events and is typified by abnormalities in left ventricular wall motion, often mimicking the clinical presentation of coronary artery disease.

**Case introduction:**

This report presents a rare case of Takotsubo cardiomyopathy (TTS) triggered by envenomation from *P. mucrosquamatus*. Following the snakebite, the patient rapidly developed severe pain and bleeding at the bite site, and subsequently experienced acute chest tightness and chest pain during hospitalisation. To elucidate the aetiology, the patient underwent a series of investigations, including electrocardiography, transthoracic echocardiography, and coronary angiography, which ultimately confirmed the diagnosis of TTS. The patient received comprehensive treatment comprising administration of anti-venom serum to neutralise the venom, fluid resuscitation, and antiplatelet therapy. The clinical condition gradually stabilised, and the patient was eventually discharged in good health.

**Conclusion:**

*Protobothrops mucrosquamatus* envenomation may precipitate Takotsubo cardiomyopathy (TTS). Given the considerable overlap in early clinical presentation between TTS and acute myocardial infarction (AMI), early utilisation of echocardiography, coronary angiography, and fractional flow reserve (FFR) assessment is crucial for accurate diagnosis. The cornerstone of treatment lies in the prompt and adequate administration of anti-venom serum, combined with fluid resuscitation and supportive symptomatic care. The judicious use of antiplatelet agents after restoration of coagulation function is generally considered safe and does not significantly increase the risk of bleeding.

## Introduction

*Protobothrops mucrosquamatus* is a medium-sized venomous snake belonging to the family Viperidae, genus Protobothrops. It is primarily distributed throughout Southeast Asia (1). Its venom is compositionally complex, containing both haemotoxins and neurotoxins, which can induce severe systemic damage (2). The typical clinical manifestations of *P. mucrosquamatus* envenomation include intense pain, local tissue necrosis, coagulopathy, and thrombocytopaenia, with some patients developing shock or even multi-organ failure (3).

Takotsubo cardiomyopathy (TTS), also known as stress-induced cardiomyopathy, is characterised by transient systolic and diastolic dysfunction of the left ventricle. Clinically, TTS mimics acute myocardial infarction, presenting with chest pain and dyspnoea; however, coronary angiography typically reveals no significant coronary obstruction (4). TTS is commonly precipitated by intense emotional or physical stressors, such as acute illness, severe pain, or serious infections (5). In recent years, the role of trauma and toxins in the pathogenesis of TTS has garnered increasing attention. Nevertheless, cases of TTS secondary to *P. mucrosquamatus* envenomation remain exceedingly rare, with no definitive reports to date (6, 7).

Herein, we report a rare case of TTS induced by *P. mucrosquamatus* envenomation. By exploring the potential pathophysiological mechanisms, imaging characteristics, and management strategies, this case aims to enhance clinical awareness and understanding of this uncommon complication.

## Case report

**Figure 1:**
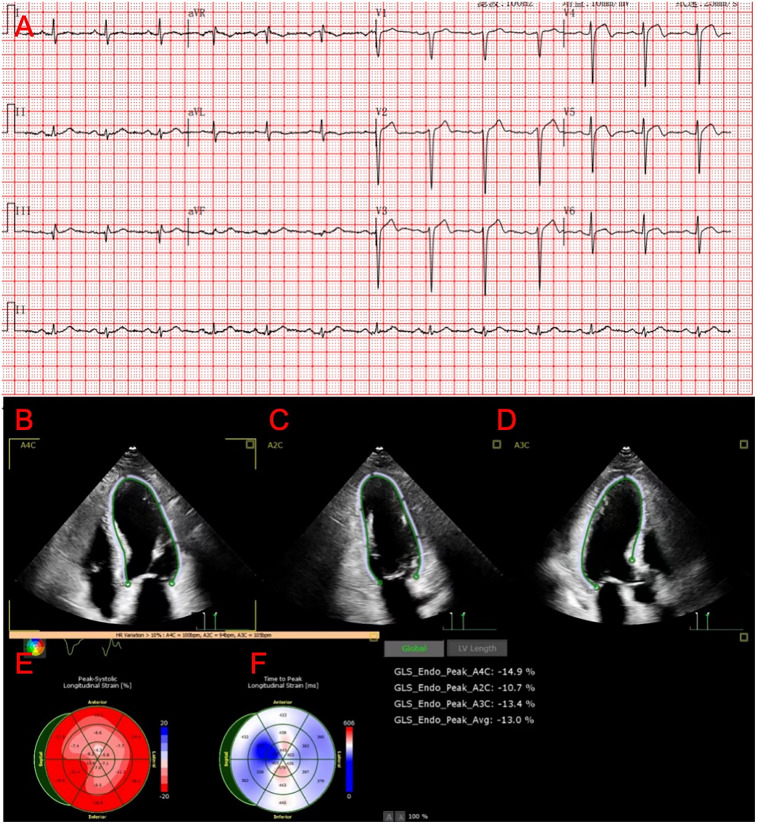
Results of the patient's ECG and ECHO. **(A)** ST-segment changes in leads V3–V6. **(B–D)** Ultrasound images of the patient's heart in the 2-chamber, 3-chamber, and 4-chamber views. **(E,F)** Graph of the Peak-Systolic Longitudinal Strain of the heart.

**Figure 2:**
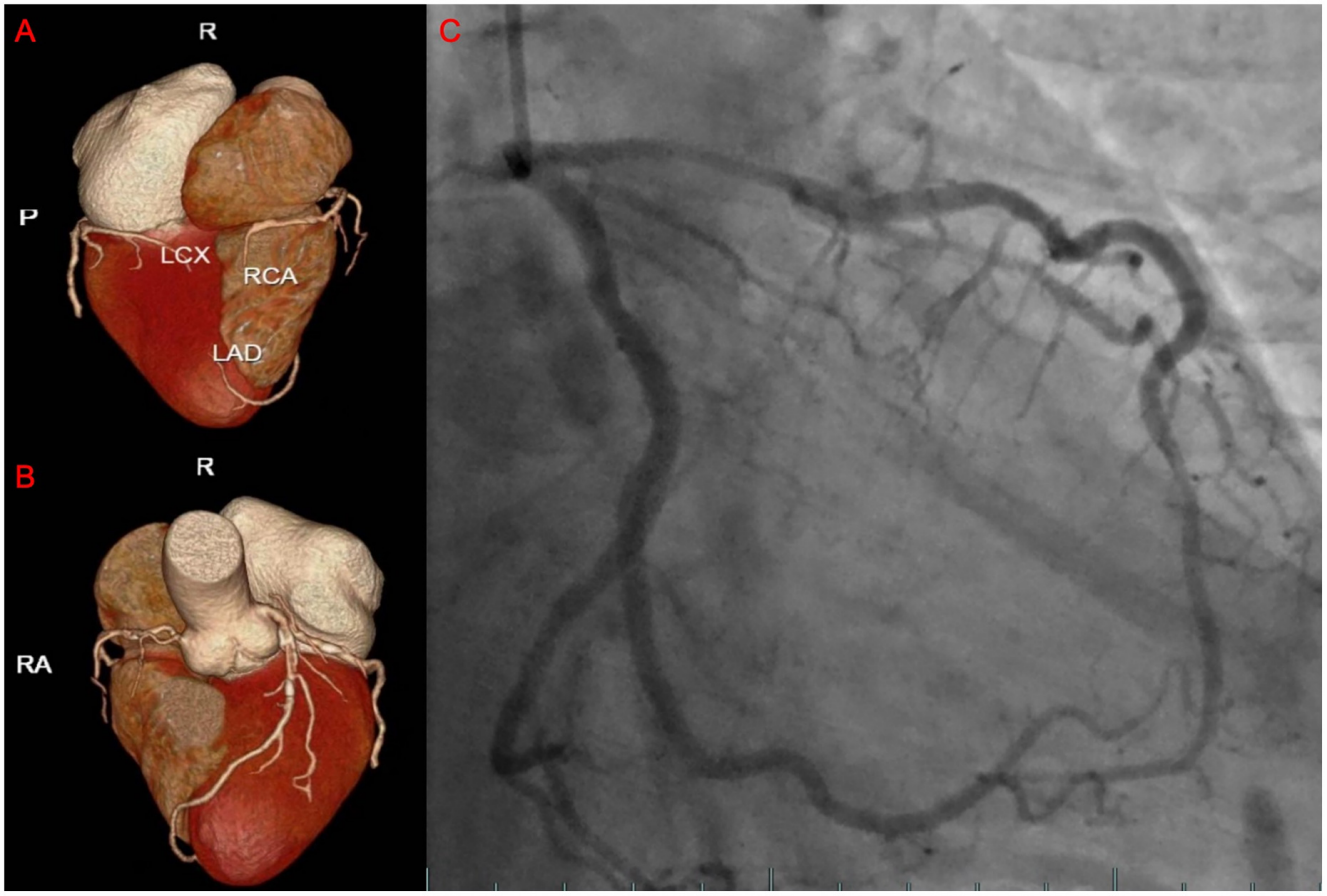
The results of the patient's coronary CTA and coronary angiography. **(A,B)** The three-dimensional reconstructed images of the patient's coronary arteries. **(C)** The images showing the passage of contrast agent through the patient's coronary arteries during angiography.

The patient is a 76-year-old male with a history of hypertension and no other known comorbidities. He was admitted to the hospital due to right ankle pain lasting 5 h following a snakebite. The patient reported that approximately 5 h prior, while fetching water near a pond, he was bitten on the right ankle by a *Protobothrops mucrosquamatus*. He subsequently developed severe pain, swelling, and localised warmth at the bite site. The patient performed self-administered first aid measures, including rinsing the wound with clean water, applying a proximal tourniquet, and ingesting Tetrastigma hemsleyanum, before presenting to the emergency department, where he was admitted with a diagnosis of venomous snakebite. Initial laboratory investigations revealed: white blood cell count 10.5 × 10^9^/L, haemoglobin 122 g/L, platelet count 178 × 10^9^/L, fibrinogen 2.19 g/L, prothrombin time (PT) 22.50 s, activated partial thromboplastin time (APTT) 42.30 s, creatine kinase (CK) 245 U/L, CK-MB 2.68 ng/ml, myoglobin 275.8 ng/ml, and troponin I 0.023 ng/ml. Thromboelastography indicated: R time of 12.8 min, K time of 4.1 min, alpha angle of 45.2°, and a maximum amplitude (MA) of 42.0 mm. Platelet aggregation test: maximum aggregation rate induced by ADP was 55%, and by AA was 51%. Physical examination showed the patient to be conscious, alert, and in good general condition. Cardiac examination revealed a regular rhythm without murmurs, clear breath sounds bilaterally without rales, and a soft, non-tender abdomen without rebound tenderness. Local examination revealed mild swelling and increased skin temperature of the left ankle, with palpable dorsalis pedis pulses. Upon admission, the patient was immediately treated with intravenous infusion of 8,000 U of anti-Deinagkistrodon antivenom serum (Shanghai Serum), intramuscular injection of tetanus vaccine and tetanus immune globulin, along with symptomatic treatments including local wound care, catharsis, diuresis, anti-oedema, and analgesia. On the second day of admission, although the swelling of the left ankle showed improvement, the patient suddenly developed chest tightness and discomfort. Bedside electrocardiography (ECG) revealed ST-segment changes in leads V3–V6 (Figure 1A), and transthoracic echocardiography demonstrated marked hypokinesis of the apical wall, paradoxical motion of the interventricular septum and left ventricular posterior wall, with impaired left ventricular systolic function (end-diastolic volume 98.01 ml, end-systolic volume 60.27 ml, ejection fraction 40%) (Figures 1B–F). Laboratory tests showed: PT 20.5 s, APTT 22.1 s, white blood cell count 21.5 × 10^9^/L, red blood cell count 3.77 × 10^12^/L, haemoglobin 117 g/L, platelet count 151 × 10^9^/L, CK 278 U/L, CK-MB 4.68 ng/ml, myoglobin 494.6 ng/ml, and troponin I 1.080 ng/ml. Given the strong suspicion of acute myocardial infarction (AMI), a chest pain workup was urgently completed, revealing focal mixed plaques in the proximal right coronary artery and mid-segment of the left anterior descending artery, with moderate-to-severe luminal stenosis (Figures 2A,B). Following consultation with cardiology, it was recommended to dynamically monitor coagulation function, cardiac enzymes, and ECG changes, and to proceed with coronary angiography as soon as clinically feasible. Antiplatelet therapy with aspirin and clopidogrel was initiated, alongside atorvastatin for plaque stabilisation. However, echocardiography demonstrated an apical ballooning pattern resembling the classical “takotsubo” appearance, and TTS could not be definitively excluded. Considering the recent history of snakebite, with preserved platelet count and coagulation profile indicating a low bleeding risk, and after obtaining informed consent from the patient's family, antiplatelet therapy was continued with close monitoring of haematological parameters. By the fourth day of admission, the patient reported significant alleviation of chest tightness. Serial ECGs, complete blood counts, coagulation profiles, and cardiac biomarkers showed no further abnormalities (Table 1), and the snakebite wound remained intact without necrosis. The patient was subsequently transferred to the cardiology department for coronary angiography.

**Table 1:** The patient's coagulation profile, cardiac enzyme profile, and complete blood count.

Parameter	Hospitalization day				
	Day 1	Day 2	Day 4	Day 6	Day 8
Cardiac enzyme profile					
Myoglobin (ng/ml)	275.8	496.6	563	351	124
Troponin (ng/ml)	0.023	1.080	1.217	0.738	0.043
CK-MB (ng/ml)	2.68	4.68	5.75	3.12	1.13
Coagulation function					
Fibrinogen (g/L)	2.19	1.98	2.23	2.42	2.18
APTT (s)	42.3	22.1	24.5	23.7	36.4
PT (s)	22.50	18.5	12.1	12.5	14.7
Complete blood count					
Haemoglobin (g/L)	122	117	121	127	130
Platelet count (×10^9^/L)	178	151	148	168	172

**Figure 3:**
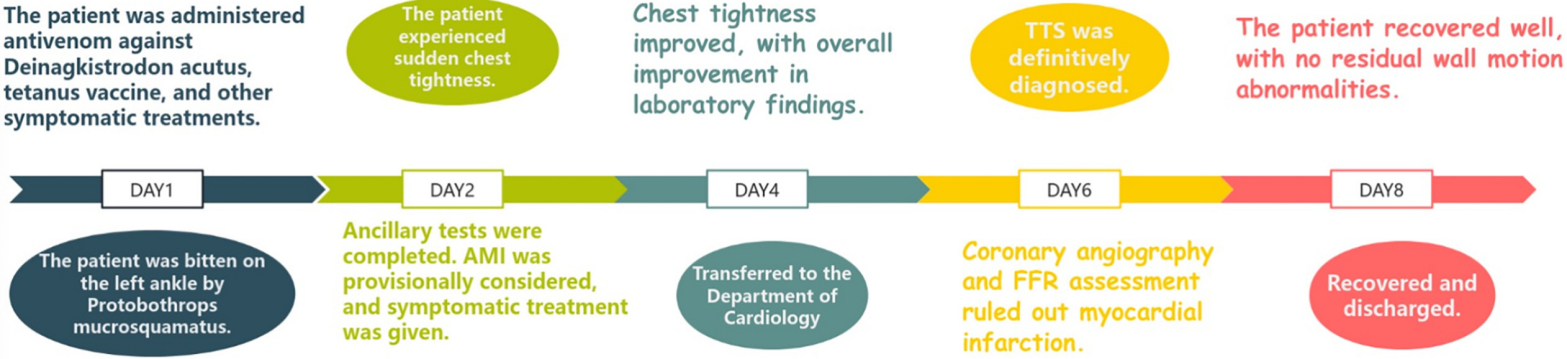
Patient's treatment course.

On the sixth day, coronary angiography demonstrated no significant stenosis in the left main coronary artery; diffuse stenosis of the proximal and mid-segments of the left anterior descending artery, with maximal stenosis of 75% (Figure 2C); no significant stenosis in the left circumflex artery; and a small right coronary artery without notable narrowing. Fractional flow reserve (FFR) assessment of the left anterior descending artery yielded an FFR of 0.84, with TIMI grade 3 distal flow. Repeat echocardiography revealed an improved ejection fraction of 69% (3D EF 68%, FS 39%), with no segmental wall motion abnormalities in the interventricular septum or left ventricular free wall, confirming the final diagnosis of TTS. By the eighth day of admission, the patient's condition remained stable, and he was discharged in good health. Post-discharge, the patient was advised to adhere to long-term secondary prevention strategies for coronary artery disease. Follow-up assessments were conducted at 3, 6, and 12 months after the patient's discharge, during which the patient remained clinically stable with no evident sequelae. Figure 3 is a timeline of the patient's clinical progression and major treatments.

## Discussion

In this case, the patient was admitted due to envenomation by *Protobothrops mucrosquamatus* and promptly received anti-venom therapy upon admission. However, during the course of treatment, he developed acute chest tightness, initially raising suspicion of acute myocardial infarction (AMI). A comprehensive diagnostic workup ultimately confirmed the diagnosis of TTS. To date, the pathophysiological mechanisms underlying TTS are not fully elucidated. The prevailing hypothesis suggests that acute stress stimuli activate the central nervous system, leading to hyperactivation of the sympathetic nervous system and a consequent surge in catecholamine levels. Excessive catecholamines, acting through β-adrenergic receptors, may induce myocardial injury. Additionally, catecholamine-induced calcium overload in cardiomyocytes may further disrupt cellular metabolism. Coronary microvascular spasm and endothelial dysfunction have also been implicated, contributing to myocardial ischaemia and precipitating the onset of TTS (8).

For patients presenting with myocardial dysfunction following *Protobothrops mucrosquamatus* envenomation in this case, it is necessary to differentiate whether the underlying cause is direct cardiotoxicity produced by venom components, or TTS induced by the intense stress response triggered by the snakebite event itself. Snakebite may not only introduce toxins that directly affect the heart or vascular system, but the event itself also constitutes a significant physiological and psychological trauma. This traumatic experience is often accompanied by severe pain and extreme fear, sufficient to trigger intense sympathetic nervous system activation, leading to a sharp elevation of endogenous catecholamine levels, such as epinephrine and norepinephrine. This abrupt catecholamine “storm” is widely recognized as one of the core pathophysiological mechanisms for inducing typical stress cardiomyopathy (8). This is consistent with the pathogenesis of TTS cases triggered by venomous bites documented in the literature (9).

On the other hand, the direct effects of *Protobothrops mucrosquamatus* venom itself cannot be overlooked. Its venom contains multiple active components, such as PLA_2_, snake venom metalloproteinases (SVMPs), thrombin-like enzymes (TLEs), and potential neurotoxins. These components have been demonstrated to induce cardiac injury through various mechanisms, including direct damage to cardiomyocytes, affecting vascular tone, interfering with coagulation, or triggering local and systemic inflammatory responses. Such effects may similarly lead to myocardial dysfunction resembling TTS or directly contribute to its development (10). Specifically, SVMPs, as key components of viperid venom, can degrade vascular basement membrane structures, directly compromising microvascular integrity, weakening endothelial cell junctions, and resulting in increased vascular permeability and local or even systemic haemorrhage (11, 12). There have been reports indicating that such damage may involve the myocardial microcirculation, thereby leading to myocardial injury (13). Meanwhile, PLA_2_ in snake venom exhibits direct cardiotoxic effects, contributing to reduced myocardial contractility and arrhythmias. It may also damage endothelial cells by hydrolysing membrane phospholipids, inducing inflammatory responses, and potentially generating excessive reactive oxygen species (12). Further studies have suggested that PLA_2_ can induce systemic bleeding tendencies and capillary damage, which may likewise involve the myocardial microcirculation and result in myocardial injury (14). The combined effects of these toxins may lead to acute endothelial dysfunction, microvascular injury, and a pro-inflammatory state. Notably, endothelial dysfunction has been widely recognised as a central mechanism in the pathophysiology of TTS (15). The severe physiological and psychological stress induced by snakebite envenomation triggers a massive release of catecholamines, which act upon an already compromised vascular bed, thereby exacerbating microvascular spasm and dysfunction. We therefore hypothesise that the direct toxic effects of snake venom on endothelial cells and the microvasculature, together with the intense catecholaminergic response induced by envenomation-related stress, jointly contributed to the onset of TTS in this patient. Nevertheless, due to the limitations inherent in this single case, it remains difficult to precisely delineate the relative contributions of venom-induced toxicity vs. stress response in the development of TTS. A complex synergistic interaction between the two is suspected.

TTS is a reversible myocardial dysfunction that closely mimics the clinical presentation of AMI. Patients may exhibit acute onset chest pain, ST-segment elevation on ECG, and elevated cardiac biomarkers, posing significant diagnostic challenges. It is currently believed that coronary microcirculatory dysfunction, manifesting as coronary and microvascular spasm, plays a key role in TTS pathophysiology. Differentiation can be achieved through assessment of coronary flow reserve (CFR) and index of microcirculatory resistance (IMR) (16). Echocardiography (ECHO) is widely regarded as the most accessible and essential tool for diagnosing TTS (17). However, in patients with TTS coexisting with coronary artery disease, echocardiography alone may not be sufficient to establish the diagnosis. In this case, the patient's ECG demonstrated ST-segment changes in leads V3–V5, coronary CTA revealed lesions in the left anterior descending (LAD) artery, and echocardiography showed apical wall motion abnormalities, all highly suggestive of AMI. Notably, the characteristic apical wall hypokinesis and reduced left ventricular ejection fraction (EF) strongly supported a diagnosis of TTS. Once the patient's condition stabilised, coronary angiography (CAG) and fractional flow reserve (FFR) assessment were promptly performed to evaluate the functional significance of the coronary lesions. FFR is a validated functional index used to determine whether coronary stenosis results in myocardial ischaemia, with an FFR ≤0.80 indicating ischaemia requiring revascularisation, whereas an FFR >0.80 suggests preserved coronary perfusion (18). In this case, the patient's FFR measurement of the LAD was 0.84, indicating adequate coronary flow despite focal stenosis, further supporting the diagnosis of TTS. Additionally, review of the patient's dynamic cardiac biomarker profiles and serial echocardiography revealed only mild elevations in cardiac enzymes, differing from the significant elevations typically observed in AMI. Rapid recovery of cardiac function and resolution of wall motion abnormalities on follow-up echocardiography further distinguished TTS from AMI (15).

Venomous snakebites are frequently associated with coagulopathy, particularly venom-induced consumption coagulopathy (VICC), which is most commonly seen in viperid envenomation. VICC presents with features resembling disseminated intravascular coagulation (DIC), including depletion of clotting factors (e.g., hypofibrinogenaemia), thrombocytopaenia, and a marked bleeding tendency (19). Under such conditions, the administration of antiplatelet agents such as aspirin and clopidogrel undoubtedly increases the risk of bleeding. Nevertheless, several case reports have demonstrated that patients who received timely anti-venom therapy and recovered coagulation function were able to undergo successful percutaneous coronary intervention (PCI), receiving standard antithrombotic regimens, including dual antiplatelet therapy (DAPT) and heparin, without major haemorrhagic complications (20). In this case, as the patient's platelet aggregation test had already shown significant improvement prior to the administration of antiplatelet therapy, no severe haemorrhagic complications occurred during hospitalisation or throughout the follow-up period. The rapid recovery of cardiac function was likely attributable to prompt management of stress triggers (both venom-related and psychological), combined with supportive treatments such as fluid resuscitation and symptomatic care. In conclusion, the management of venomous snakebite complicated by acute cardiovascular events requires close collaboration between toxicology and cardiology teams. Individualised treatment strategies should be guided by the patient's coagulation status and myocardial injury severity.

As a case report, this study has limitations. The findings may not be generalizable, and a direct causal relationship cannot be definitively established without larger studies.

## Conclusion

Envenomation by *Protobothrops mucrosquamatus* may precipitate TTS. Early utilisation of echocardiography, coronary angiography, and fractional flow reserve (FFR) assessment is instrumental in establishing the diagnosis. Adequate administration of anti-venom serum combined with supportive therapy constitutes the cornerstone of treatment. Once coagulation function is restored, the judicious use of antiplatelet or anticoagulant therapy typically does not significantly increase the risk of bleeding.

## Data Availability

The original contributions presented in the study are included in the article/Supplementary Material, further inquiries can be directed to the corresponding author.
